# Clinical outcomes of ramucirumab plus docetaxel in the treatment of patients with non-small cell lung cancer after immunotherapy: a systematic literature review

**DOI:** 10.3389/fonc.2023.1247879

**Published:** 2023-09-04

**Authors:** Edward B. Garon, Carla Visseren-Grul, Maria Teresa Rizzo, Tarun Puri, Suresh Chenji, Martin Reck

**Affiliations:** ^1^ David Geffen School of Medicine, University of California, Los Angeles/Translational Research in Oncology-United States Network, Los Angeles, CA, United States; ^2^ Eli Lilly and Company, Lilly Corporate Center, Indianapolis, IN, United States; ^3^ Eli Lilly and Company, Bengaluru, Karnataka, India; ^4^ Department of Thoracic Oncology, Airway Research Center North (ARCN), Member of the German Center for Lung Research (DZL), Lung Clinic Grosshansdorf, Großhansdorf, Germany

**Keywords:** non-small cell lung cancer, ramucirumab, docetaxel, immune checkpoint inhibitors, antiangiogenic therapy

## Abstract

**Introduction:**

In the REVEL trial, ramucirumab plus docetaxel demonstrated significant improvements in overall survival (OS), progression-free survival (PFS), and overall response rate (ORR) compared with placebo plus docetaxel for treatment of metastatic non-small cell lung cancer (NSCLC) that progressed during or after platinum-based chemotherapy. Since the approval of ramucirumab plus docetaxel, immune checkpoint inhibitors (ICIs), either as single agents or in combination with chemotherapy, have become the standard of care for first-line treatment of patients with advanced NSCLC. However, efficacy and safety data for ramucirumab plus docetaxel after prior ICI treatment from randomized controlled clinical studies are lacking.

**Methods:**

Following Preferred Reporting Items for Systematic Reviews and Meta-Analyses guidelines, a systematic literature review was performed. Electronic databases and select international oncology conference proceedings were searched. Studies published between 01 January 2014 and 01 July 2022, which evaluated 2 efficacy outcomes (and included at least 1 time-to-event endpoint) or safety outcomes of ramucirumab plus docetaxel in NSCLC that progressed after prior ICI treatment, were identified. Twelve studies were included in the analysis. Two treatment groups were selected: ramucirumab plus docetaxel after prior ICI ± chemotherapy (RAM + DTX ICI pre-treated) and ramucirumab plus docetaxel after prior chemotherapy only (RAM + DTX ICI naïve). OS, PFS, ORR, disease control rate (DCR), and safety data were extracted and descriptively summarized across both treatment groups.

**Results:**

The pooled weighted median PFS and median OS were 5.7 months (95% confidence interval [CI]: 3.9-6.8) and 11.2 months (95% CI: 7.5-17.5), respectively, in the RAM + DTX ICI pre-treated group and 3.8 months (95% CI: 2.3-4.1) and 13.5 months (95% CI: 8-24.0), respectively, in the RAM + DTX ICI naïve group. The ORR and DCR ranged from 20.9% to 60.0% and from 62.4% to 90.0%, respectively, in the RAM + DTX ICI pre-treated group and from 17.7% to 20.0% and from 57.1% to 75.0%, respectively, in the RAM + DTX ICI naïve group. The safety profile across studies was consistent between both treatment groups, and no new safety signals were reported.

**Conclusions:**

Cumulatively, these results support the combination of ramucirumab plus docetaxel as an effective and safe subsequent therapy for the treatment of patients with metastatic NSCLC with disease progression irrespective of previous ICI treatment.

## 1 Introduction

The introduction of immune checkpoint inhibitors (ICIs) for the first-line treatment of patients with advanced non-small cell lung cancer (NSCLC) without driver alterations has dramatically improved clinical outcomes, with patients experiencing prolonged overall survival (OS) and durable responses compared with chemotherapy alone (1–6). Several ICIs, including anti-programmed death 1 (PD-1) or anti-programmed death ligand 1 (anti-PD-L1) antibodies, are currently recommended for the front-line treatment of metastatic NSCLC, either as single agents in select patient populations or in combination with chemotherapy or other immunotherapeutic agents (7, 8). However, approximately 50% of patients receive subsequent treatment upon progression during or after first-line treatment (9–12).

The current treatment guidelines for patients with metastatic NSCLC who experience disease progression after standard-of-care therapy in the first-line setting (7, 8) consist of single-agent chemotherapy or a combination of docetaxel with an antiangiogenic agent such as ramucirumab and nintedanib, or single agent anti-PD-(L)1 antibodies if not previously administered (13–17). Clinical outcomes with single-agent chemotherapy are modest. Treatment with docetaxel, in comparison to best supportive care, resulted in an overall response rate (ORR) of 7.1%, time to progression of 10.6 weeks, and median OS of 7.0 months (18). Similarly, treatment with gemcitabine, pemetrexed, or *nab*-paclitaxel demonstrated a median OS of 5.1 months (19), 8.3 months (20), and 8.5 months (21), respectively. Combination approaches with chemotherapy and antiangiogenic agents in the second-line setting have produced more favorable outcomes compared with chemotherapy alone.

Ramucirumab is a fully human immunoglobulin G1 monoclonal antibody that specifically binds to the vascular endothelial growth factor (VEGF) receptor-2 extracellular domain with high affinity, preventing binding of all VEGF ligands and subsequent receptor activation (22). In the phase 3 REVEL trial, the combination of ramucirumab plus docetaxel demonstrated a significant improvement in median OS (10.5 vs 9.1 months; hazard ratio [HR]: 0.86; *P*=0.023), median progression-free survival (median PFS, 4.5 vs 3.0 months; HR: 0.76; *P*<0.0001), and ORR (23% vs 14%; odds ratio: 1.89; *P*<0.0001) relative to docetaxel plus placebo in patients with stage IV NSCLC whose disease had progressed during or after first-line platinum-based chemotherapy (13). Importantly, ramucirumab plus docetaxel had a manageable safety profile and no detrimental impact on quality of life (13, 23). Based on these results, ramucirumab in combination with docetaxel received regulatory approval in 2014 in the United States and European Union for the treatment of patients with metastatic NSCLC with disease progression during or after platinum-based chemotherapy (24, 25). Additionally, second-line treatment with ramucirumab plus docetaxel resulted in an improvement of median PFS relative to docetaxel in other studies (26, 27), including in a randomized phase 2 trial that enrolled 160 Japanese patients with stage IV NSCLC (median PFS: 5.2 vs 4.2 months; HR: 0.83, 95% CI: 0.59-1.16) (28).

Studies have also demonstrated improved efficacy with other antiangiogenic agents in combination with chemotherapy in the second-line treatment of metastatic NSCLC. In the LUME-Lung 1 study, the combination of nintedanib, an antiangiogenic agent targeting 3 angiogenesis-related transmembrane receptors (14), with docetaxel resulted in statistically significant improvement in PFS, compared to docetaxel monotherapy. However, a statistically significant improvement in OS was observed only in the subgroup of patients with adenocarcinoma histology but not in the intention-to-treat population (13, 14). Hence, the approval of nintedanib in the European Union was restricted to NSCLC patients with adenocarcinoma histology who have undergone first-line chemotherapy (29).

The currently recommended treatment options for patients with NSCLC whose disease progressed during or after first-line treatment were investigated before the approval of ICIs in immunotherapy-naïve patients. Therefore, the results from these trials, including REVEL, do not optimally reflect the current patient population with disease progression after ICI treatment. Randomized controlled studies investigating the efficacy and safety of ramucirumab plus docetaxel in the post-immunotherapy setting are lacking. Nevertheless, the efficacy and safety of ramucirumab plus docetaxel in patients previously treated with ICIs have been reported in recent years, mostly from retrospective observational studies (30–44) and electronic health record studies (45–47). We conducted a systematic literature review (SLR) to consolidate the available evidence on the efficacy and safety of ramucirumab plus docetaxel when administered to patients with metastatic NSCLC previously treated with ICIs.

## 2 Methods

### 2.1 Search strategy

The SLR search, selection, and data extraction were conducted and reported using the Preferred Reporting Items for Systematic Reviews and Meta-Analyses 2020 statement (48).

PubMed and EMBASE were searched to identify English-language manuscripts and abstracts submitted to select international oncology meetings (American Society of Clinical Oncology, European Society for Medical Oncology and World Conference on Lung Cancer) between 01 January 2014 and 01 July 2022. This literature search was completed on 11 July 2022. The following MeSH terms were used: (“non-small cell lung cancer” OR “NSCLC”) AND (“docetaxel” OR “DTX”) AND (“ramucirumab” OR “RAM”) AND (“immunotherapy” OR “Immune checkpoint inhibitor” OR “nivolumab” OR “pembrolizumab” OR “atezolizumab” OR “anti PD-1” OR “PD-L1”) (**Supplementary Text 1.1**).

The analysis included studies that evaluated at least 2 efficacy endpoints with at least one being a time-to-event endpoint (PFS or OS) of treatment with ramucirumab plus docetaxel in patients with advanced NSCLC who received prior ICI treatment. Studies reporting safety outcomes (irrespective of whether they reported efficacy outcomes) were also included. Two treatment groups were selected: ramucirumab plus docetaxel after prior ICI ± chemotherapy (RAM + DTX ICI pre-treated) and ramucirumab plus docetaxel after prior chemotherapy only (RAM + DTX ICI naïve).

The studies differed from each other on account of variations in several characteristics including sample size, lines of prior treatment, presence of driver alterations, PD-L1 expression, and patient performance status. Individual patient-level data was not available from any study. Therefore, data from the included studies were analyzed in a descriptive manner without formal statistical analysis. PFS, OS, ORR, disease control rate (DCR), and safety results were extracted and summarized for the 2 treatment groups.

### 2.2 Statistical analysis

Efficacy outcomes for each treatment group were pooled from the studies selected for efficacy analysis and compared descriptively. Estimates of median PFS and median OS from individual studies were pooled using the weighted median of medians, and approximate 95% confidence intervals (CIs) for weighted pooled medians were calculated using the wtd.quantile function from the Hmisc package of the R Statistical Software (v4.1.2; R Core Team 2021) (49). The weighted median of the study-specific medians was a pooled median estimate, where the weights were proportional to the number of patients in the study because sample sizes in the studies were independent of the individual study medians. ORR and DCR were summarized as ranges of percentages with 95% CIs from individual studies. For studies with available safety data, a descriptive comparison was performed for common adverse events (AEs) and AEs of special interest.

### 2.3 Assessment of bias

The Newcastle-Ottawa Scale was used to assess the risk of bias of studies comparing RAM + DTX ICI pre-treated versus RAM + DTX ICI naïve treatment groups (50). The Newcastle-Ottawa Scale includes 8 items within 3 categories: selection (4 items, 1 point each), comparability (1 item, up to 2 points), and outcome (3 items, 1 point each). The sum of points represents the methodologic quality of each study included in the SLR, with 9 points indicating the highest quality and 0 points the lowest quality. For studies that reported results from the RAM + DTX ICI pre-treated group only, the “quality assessment tool for before-after (pre-post) studies with no control group” outlined by the National Institutes of Health was used to assess the risk of bias (51). This tool comprises 12 questions, such as selection, reporting, or observer bias, with responses of yes, no, other, not reported (NR), not applicable, or cannot determine. All selected studies were free from the risk of bias as per the Newcastle-Ottawa Scale (**Supplementary Table 1**) and National Institutes of Health tools score (**Supplementary Table 2**). Two independent reviewers from Eli Lilly and Company assessed the risk of bias in the included studies for the RAM + DTX ICI pre-treated versus RAM + DTX ICI naïve groups. Disagreements were resolved by consensus with assistance from a third reviewer, also from Eli Lilly and Company.

## 3 Results

### 3.1 Search results

**Figure 1:**
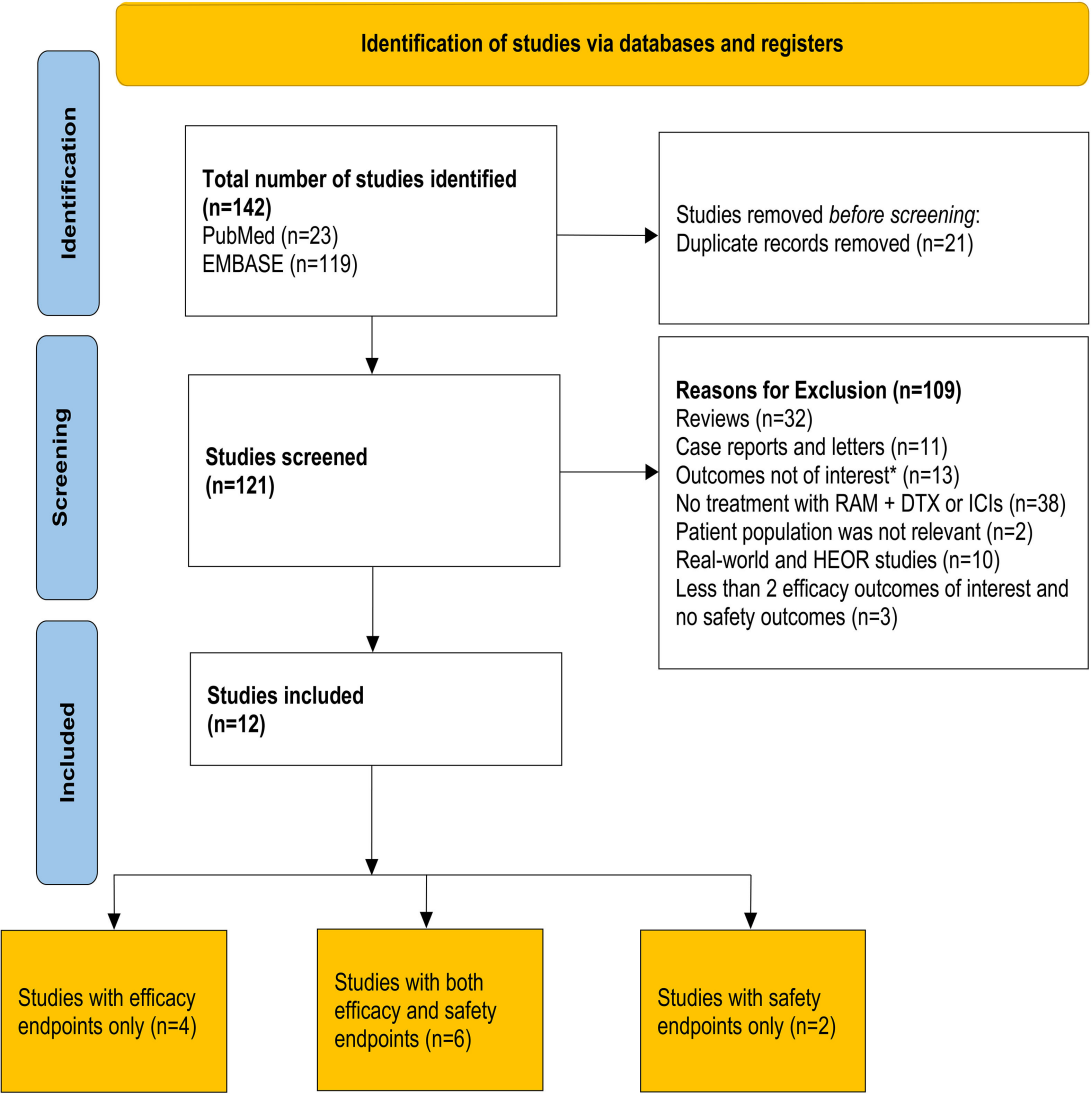
PRISMA flow diagram for article selection. The Preferred Reporting Items for Systematic Reviews and Meta-Analyses (PRISMA) flow diagram for the systematic review, detailing the database searches and the number of studies screened and included for final analysis. *Outcomes that were not of interest included 12-month overall survival rate and time to treatment discontinuation. DTX, Docetaxel; HEOR, Health economics and outcomes research; ICI, Immune checkpoint inhibitor; n, Number of patients; RAM, Ramucirumab.

An initial literature search yielded 142 entries, with 23 results from PubMed and 119 results from EMBASE (**Figure 1**). Twelve of the 142 studies were included in the final analysis. Of these, 10 were retrospective observational studies (30–34, 36, 38–41) and 2 were prospective studies (43, 44). Among the 10 retrospective studies, 6 studies reported both safety and efficacy endpoints (30, 32–34, 39, 40) and 4 studies reported only efficacy endpoints (31, 36, 38, 41). Among the 10 studies included in the efficacy analysis, 5 compared outcomes between RAM + DTX ICI pre-treated and RAM + DTX ICI naïve patients (30–32, 38, 41) while the remaining 5 studies reported efficacy results only for RAM + DTX ICI pre-treated patients (33, 34, 36, 39, 40). Safety outcomes were extracted from 6 retrospective studies and 2 prospective studies (30, 32–34, 39, 40, 43, 44). Of the 2 prospective studies, one was a phase 2 randomized clinical trial that investigated the combination of pembrolizumab plus ramucirumab in patients whose disease had progressed during or after front-line ICI and platinum-based chemotherapy. The control arm, which comprised investigator’s choice standard-of-care therapy, included ramucirumab plus docetaxel among other agents. Safety data from the cohort of patients treated with ramucirumab plus docetaxel were used for the safety analysis reported in this SLR (44). The other prospective trial was a single-arm, multicenter, post marketing study, which reported safety and a time-to-event efficacy endpoint (12-month OS rate) in patients treated with ramucirumab plus docetaxel in the post-immunotherapy setting only (43).

Eight studies were conducted in Japan where docetaxel doses ranged from 60 to 75 mg/m^2^ (30–32, 38–41, 43), while 2 studies each were conducted in Germany (33, 34), and in the United States (36, 44), where docetaxel is generally administered at a dose of 75-mg/m^2^.

Anti-PD-(L)1 agents use varied across studies, with nivolumab (21.7%), pembrolizumab (3.1%) and atezolizumab (2.8%), being the most frequently reported single-agent ICIs (61.0%) (30, 32, 34, 38, 39, 41). Chemoimmunotherapy combinations were used in 27.9% of patients (30, 33, 38, 40). The most commonly used combination of platinum-based chemotherapy and ICIs was platinum plus pemetrexed plus pembrolizumab (16.0%) (33, 40). Of the 12 studies, 4 included patients who had received only previous ICI monotherapy (32, 34, 39, 41), 2 included patients who had received only previous ICI in combination with chemotherapy (33, 40), 2 studies included patients who had received ICI in combination with chemotherapy or ICI monotherapy (30, 38) and the other 4 studies did not specify the type of immunotherapy used (31, 36, 43, 44). Of note, 2 studies that included patients who had received only previous ICI monotherapy investigated ramucirumab plus docetaxel in the third-line setting (32, 34), while both studies that included patients who had received only previous ICI in combination with chemotherapy investigated ramucirumab plus docetaxel in the second-line setting (**Table 1**) (33, 40). The other 8 studies did not specify the treatment line for ramucirumab plus docetaxel. Median follow-up was not reported for most studies but when reported ranged from 5.0 months (33) to 17.9 months (44).

**Table 1:** Study characteristics.

Study (reference)	Study type	Country	Treatment	No. of total patients		Type of prior ICI (no. of patients)	Median lines of treatment (range)	Median follow-up (months)
				ICI pre-treated	ICI naïve			
RAM + DTX ICI pre-treated vs ICI naïve								
Harada, 2019 (30)^1^	Retrospective, single-center, observational, 2 treatment arms	Japan	RAM + DTX ICI pre-treated vs naïve	18	21	Nivo (11), Pembro (1), Atezo (1), ICI + Chemo (5)^2^	3 (2–5)	NS
Yoshimura, 2019 (31)	Retrospective, multicenter, observational, 2 treatment arms	Japan	RAM + DTX ICI pre-treated vs naïve	52	83	NS	3 (2–9)	NS
Kato, 2020 (32)^1^	Retrospective, multicenter, observational, 2 treatment arms	Japan	ICI pre-treated vs ICI naïve^3^	62	84	Nivo (NS), Pembro (NS)	3^7^	ICI pre-treated: 8.1 (95% CI, 7.5-9.4) Control cohort: 9.3 (95% CI, 8.7-9.9)
Tozuka, 2020 (41)	Retrospective, single center, observational, 4 treatment arms	Japan	RAM + DTX vs DTX ICI pre-treated vs naïve	21	25	Nivo (NS), Pembro (NS), Atezo (NS)	2/3^9^	NS
Nishimura, 2022 (38)	Retrospective, multicenter, observational, 4 treatment arms	Japan	RAM + DTX vs DTX ICI pre-treated vs naïve	17	26	Atezo (1), Nivo (4), Pembro (4), CBDCA + Nab-ptx + Atezo (1), CBDCA + Pem + Pembro (6), CBDCA + Nab-ptx + Pembro (1)	NS	NS
Chen, 2022 (43)^1^	Prospective, single center, observational, post marketing study, single treatment arm	Japan	RAM + DTX ICI pre-treated vs naïve^3^	172	226	NS	NS	12 from start of RAM + DTX^10^
RAM + DTX ICI pre-treated								
Shiono, 2019 (39)^1^	Retrospective, single-center, observational, single treatment arm	Japan	RAM + DTX ICI pre-treated	20		Nivo (20)	3 (3–5)	NS
Brueckl, 2020 (34)^1^	Retrospective, multicenter, observational, single treatment arm	Germany	RAM + DTX ICI pre-treated	67		Nivo (49), Pembro (7), Atezo (9), Durva (2)	3^7^	≥6^10^
Dawar, 2021 (36)	Retrospective, single-center, observational, single treatment arm	United States	RAM + DTX ICI pre-treated	35		NS	2–3	NS
Brueckl, 2021 (33)^1^	Retrospective, multicenter, observational, single treatment arm	Germany	RAM + DTX ICI pre-treated	77		Pt + Pem + Pembro (50), Pt + Ptx/Nab-ptx + Pembro (9), Pt + Ptx/Nab-ptx + Atezo (8), Pt + Pem + Durva + Treme (2), CBDCA + Ptx + Bev + Atezo (2), Pt + GemVin + Pembro (3), Pt + GemVin + Durva + Treme (3)	2^8^	≥5 before data cut-off
Ishida, 2021 (40)^1^	Retrospective, multicenter, observational, 2 treatment arms	Japan	RAM + DTX ICI pre-treated^4^	18		Pt + Pem + Pembro (12), CBDCA + Ptx/Nab-ptx + Pembro (3), CBDCA + Ptx + Atezo + Bev (2), CBDCA + Nab-ptx + Atezo (1)	2^8^	9 from start of second-line treatment
Reckamp, 2022 (44)^1^	Randomized, multicenter, phase 2, 2 treatment arms	United States	RAM + Pembro vs SoC ICI pre-treated^5^	136^6^		Nivo (NS), Pembro (NS), Atezo (NS), Durva (NS)	NS	17.9

^1^The study also reported safety outcomes; ^2^The types of chemotherapy were not specified; ^3^RAM + DTX was a subgroup analysis; ^4^The study also included patients who received pre-treatment with single‐agent chemotherapeutic ICIs: 11 received DTX and 4 received TS‐1; ^5^RAM + DTX ICI pre-treated was a subgroup of the Standard of Care arm; ^6^Forty-five of these patients in the SoC arm were previously treated with ramucirumab plus docetaxel and included in our pooled efficacy analysis, and 44 patients were included in our pooled safety analysis; ^7^Studies reported outcomes for patients treated with RAM + DTX in the third line only; ^8^Studies reported outcomes for patients treated with RAM + DTX in the second line only; ^9^Not specified; ^10^Reported only total duration of follow-up.

Atezo, Atezolizumab; Bev, Bevacizumab; CBDCA, Carboplatin; CI, Confidence interval; DTX, Docetaxel; Durva, Durvalumab; GemVin, Gemcitabine/vinorelbine; ICI, Immune checkpoint inhibitor; n, Number of patients; Nab-ptx, Nanoparticle albumin bound-paclitaxel; Nivo, Nivolumab; NS, Not specified; Pembro, Pembrolizumab; Pem, Pemetrexed; Pt, Platinum; Ptx, Paclitaxel; SoC, Standard of care; Treme, Tremelimumab; TS-1, 5-Fluorouracil (5-FU) 1.

### 3.2 Patient baseline characteristics

The median age of patients ranged from 59 to 70 years. Most patients had an Eastern Cooperative Oncology Group performance status score of 0 or 1 with an unknown PD-L1 status and wild-type epidermal growth factor receptor (*EGFR*). Patient characteristics across studies are summarized in **Table 2**.

**Table 2:** Patient characteristics.

Study (reference)	Total patients, n		Age, years, median (95% CI)		Male/female, n		ECOG PS 0,1/≥2, n		Never smoker/current or former smoker, n		ADC/SCC, n		*EGFR*/*ALK* alterations, n		PD-L1 status negative/positive, n	
	ICI pre-treated	ICI naïve	ICI pre-treated	ICI naïve	ICI pre-treated	ICI naïve	ICI pre-treated	ICI naïve	ICI pre-treated	ICI naïve	ICI pre-treated	ICI naïve	ICI pre-treated	ICI naïve	ICI pre-treated	ICI naïve
RAM + DTX ICI pre-treated vs naïve																
Harada, 2019 (30)^1^	18	21	67 (54–78)	65 (50–76)	15/3	10/11	17/1	18/3	2/16	7/14	13/5	20/0	2/0	8/1	4/4	6/4
Yoshimura, 2019 (31)^2^	52	83	66 (37–83)		95/40		125/10		43/92		114/21		27/2		44/38	
Kato, 2020 (32)^1^	62	84	66 (38–80)	66 (30–79)	54/23	57/37	63/6	85/5	20/57	28/65	61/12	87/6	4/2	23/3	12/26	30/24
Tozuka, 2020 (41)	21	25	65 (36–73)	59 (36–77)	17/4	10/15	20/1	24/1	4/17	11/14	17/4	22/1	0^3^	6^3^	6/7	4/8
Nishimura, 2022 (38)^2^	17	26	NS		23/20		38/5		13/30		36/5		14^8^/1		7/14	
Chen, 2022 (43)^1,2^	172	226	67 (29–88)		273/125		374/24		NS		311/66		102/12		NS	
RAM + DTX ICI pre-treated																
Shiono, 2019 (39)^1^	20	—	70 (55–77)	—	12/8	—	18/2	—	8/12	—	16/3	—	3/NS	—	NS	—
Brueckl, 2020 (34)^1^	67	—	62 (43–82)	—	46/21	—	61/2	—	NS	—	39/24	—	NS^6^	—	15/25	—
Dawar, 2021 (36)	35	—	65 (45–76)	—	18/17	—	NS	—	7/28	—	33/2	—	NS	—	NS	—
Brueckl, 2021 (33)^1^	77	—	63 (41–83)	—	53/24	—	68/5	—	NS	—	55/16	—	17^4^	—	33/37	—
Ishida, 2021 (40)^1^	18	—	69 (43–79)	—	11/7	—	16/2	—	2/16	—	16^5^/2	—	NS^6^	—	5/10	—
Reckamp, 2022 (44)^1,7^	45	—	NS	—	NS	—	NS	—	NS	—	NS	—	NS	—	NS	—

^1^The study also reported safety outcomes; ^2^Patient characteristics for these studies were only reported for the total RAM + DTX population and not separated by ICI pre-treated or naïve; ^3^Type of oncogene not specified; ^4^KRAS mutation; ^5^Nonsquamous cell carcinoma; ^6^Patients were excluded if they had EGFR activating mutations or ALK arrangement fusion; ^7^Patient characteristics were only reported for the total standard of care group, not the RAM + DTX subgroup. PD-L1 status of <1 is negative and PD-L1 status ≥1 to 50 is positive; ^8^EGFR mutations included 7 patients with an Exon 19 deletion, 6 patients with an Exon 21 L858R mutation and 1 patient with an Exon 20 insertion.

ADC, Adenocarcinoma; ALK, Anaplastic lymphoma kinase; CI, Confidence interval; DTX, Docetaxel; ECOG PS, Eastern Cooperative Oncology Group performance status; EGFR, Epidermal growth factor receptor; ICI, Immune checkpoint inhibitor; n, Number of patients; NS, Not specified; PD-L1, Programmed death ligand 1; RAM, Ramucirumab; SCC, Squamous cell carcinoma.

### 3.3 Efficacy

Ten studies were included in the final efficacy analysis. From these studies, 387 pooled patients received ramucirumab plus docetaxel after ICI treatment (RAM + DTX ICI pre-treated) (30–34, 36, 38–41) and 239 pooled patients received ramucirumab plus docetaxel after chemotherapy only (RAM + DTX ICI naïve) (30–32, 38, 41). All included studies measured efficacy outcomes from the first day of treatment with ramucirumab plus docetaxel.

#### 3.3.1 Progression-free survival

**Figure 2:**
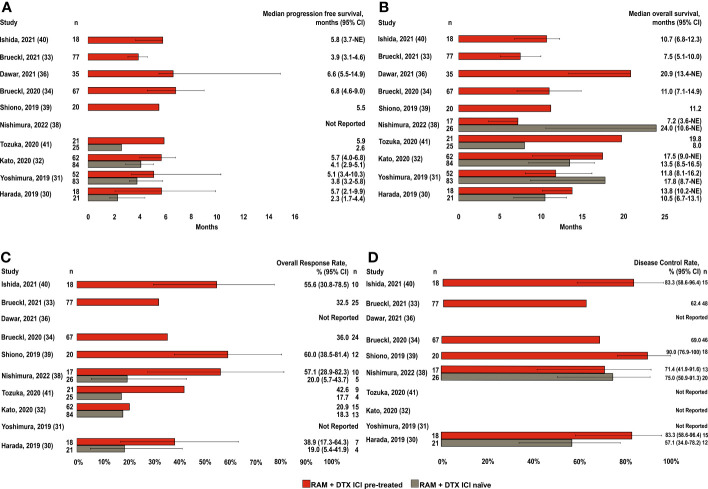
Efficacy endpoints across studies in RAM + DTX ICI pre-treated and ICI naïve patients. **(A)** Median progression-free survival; **(B)** Median overall survival; **(C)** Overall response rate; and **(D)** Disease control rate. Red bars indicate the RAM+DTX ICI pre-treated group and brown bars the RAM+DTX ICI naïve group. CI, Confidence interval; DTX, Docetaxel; ICI, Immune checkpoint inhibitor; n, Number of patients; NE, Not estimable; RAM, Ramucirumab.

Of the 10 studies included in the efficacy analysis, median PFS was reported in 9 for RAM + DTX ICI pre-treated patients (30–34, 36, 39–41) and 4 of these 9 studies also reported median PFS in RAM + DTX ICI naïve patients (**Figure 2A**) (30–32, 41).

Of the 9 studies in RAM + DTX ICI pre-treated patients, 5 included patients in the RAM + DTX ICI pre-treated group only (33, 34, 36, 39, 40). Median PFS in these 5 studies ranged from 3.9 months (95% CI: 3.1-4.6) to 6.8 months (95% CI: 4.6-9.0) (33, 34). The other 4 studies reported median PFS across both RAM + DTX ICI pre-treated and RAM + DTX ICI naïve patients (30–32, 41). All 4 studies reported a trend toward improved median PFS for RAM + DTX ICI pre-treated patients (5.1 months [95% CI: 3.4-10.3] to 5.9 months [95% CI: Not reported (NR)]) compared with RAM + DTX ICI naïve patients (2.3 months [95% CI: 1.7-4.4] to 4.1 months [95% CI: 2.6-5.8]) (30–32, 41), with statistical significance established in 2 studies (*P=*0.012 and *P*=0.041) (**Table 3**) (30, 31).

**Table 3:** Treatment comparisons between the ICI pre-treated and ICI naïve groups.

Study (reference)	Patients, n		Treatment comparison RAM + DTX ICI pre-treated vs RAM + DTX ICI naïve
	RAM + DTX ICI pre-treated	RAM + DTX ICI naïve	
Harada, 2019 (30)	18	21	**PFS** Univariate: 0.43 (0.21–0.89); *P*=0.023* Multivariate: 0.36 (0.16–0.80); *P*=0.012* **OS** Univariate: 0.46 (0.20–1.07); *P*=0.071 Multivariate: 0.41 (0.16–1.04); *P*=0.061
Yoshimura, 2019 (31)	52	83	**PFS** Univariate: 0.63 (0.39–1.02); *P*=0.059 Multivariate: 0.59 (0.35–0.98); *P*=0.041* **OS** Matched pair analysis: 1.71 (0.84–3.47), *P=*0.14
Kato, 2020 (32)	61/62	84	**PFS** IPTW adjusted: 0.97 (0.68–1.38); *P=*0.86 **OS** IPTW adjusted: 0.67 (0.41–1.11); *P=*0.12
Tozuka, 2020 (41)	21	25	—
Nishimura, 2022 (38)	17	26	**OS** *P=*0.20

Data are shown as hazard ratio (95% confidence interval) with corresponding P value, unless otherwise stated.

*Significance established.

DTX, Docetaxel; ICI, Immune checkpoint inhibitor; IPTW, Inverse probability treatment weighting; OS, Overall survival; PFS, Progression-free survival; RAM, Ramucirumab.

The pooled weighted median PFS across all 9 studies was 5.7 months (95% CI: 3.9-6.8) in the RAM + DTX ICI pre-treated group and 3.8 months (95% CI: 2.3-4.1) in the RAM + DTX ICI naïve group (**Table 4**). The median PFS ranged from 3.9 months (95% CI: 3.1-4.6) (33) to 6.8 months (95% CI: 4.6-9.0) (34) in the RAM + DTX ICI pre-treated groups and from 2.3 months (95% CI: 1.7-4.4) (30) to 4.1 months (95% CI: 2.9-5.1) (32) in the RAM + DTX ICI naïve groups.

**Table 4:** Pooled weighted efficacy outcomes.

	Progression-free survival		Overall survival	
	ICI pre-treated n= 370	ICI naïve n= 213	ICI pre-treated n= 387	ICI naïve n= 239
**Median, months (95% CI)**	5.7 (3.9–6.8)	3.8 (2.3–4.1)	11.2 (7.5–17.5)	13.5 (8.0–24.0)

CI, Confidence interval; ICI, Immune checkpoint inhibitor.

#### 3.3.2 Overall survival

Median OS was reported in all 10 studies included in the efficacy analysis. Median OS for RAM + DTX ICI pre-treated patients were reported in all 10 studies (30–34, 36, 38–41), while only 5 studies reported median OS for RAM + DTX ICI naïve patients (**Figure 2B**) (30–32, 38, 41).

Of the 5 studies that included only RAM + DTX ICI pre-treated patients (33, 34, 36, 39, 40), median OS ranged from 7.5 months (95% CI: 5.1-10.0) to 20.9 months (95% CI: 13.4-not estimable [NE]) (33, 36). The other 5 studies reported outcomes for both RAM + DTX ICI pre-treated and RAM + DTX ICI naïve patients (30–32, 38, 41). Three of these 5 studies reported longer median OS in the RAM + DTX ICI pre-treated group (13.8 months [95% CI: 10.2-NE] to 19.8 months [95% CI: NR]) compared with the RAM + DTX ICI naïve group (8.0 months [95% CI: NR] to 13.5 months [95% CI: 8.5-16.5]), but statistical significance was not established in any of these studies (30, 32, 41). In the other 2 studies, a numerically longer median OS was observed in the RAM + DTX ICI naïve group (17.8 months [95% CI: 8.7-NE] and 24.0 months [95% CI: 10.6-NE]) compared with the RAM + DTX ICI pre-treated group (11.8 months [95% CI: 8.1-16.2] and 7.2 months [95% CI: 3.6-NE]), but this difference was not statistically significant in either study (*P*=0.14 and *P*=0.20, respectively) (**Table 3**) (31, 38).

The pooled weighted median OS across all 10 studies was 11.2 months (95% CI: 7.5-17.5) in the RAM + DTX ICI pre-treated group and 13.5 months (95% CI: 8.0-24.0) in the RAM + DTX ICI naïve group (**Table 4**). The median OS ranged from 7.2 months (95% CI: 3.6-NE) (38) to 20.9 months (95% CI: 13.4-NE) (36) in the RAM + DTX ICI pre-treated groups and from 8.0 months (95% CI: NR) (41) to 24.0 months (95% CI: 10.6-NE) (38) in the RAM + DTX ICI naïve groups.

#### 3.3.3 Overall response rate

Of the 10 studies included in the efficacy analysis, ORRs were reported in 8 studies (**Figure 2C**) (30, 32–34, 38–41) in RAM + DTX ICI pre-treated patients (30, 32–34, 38–41), 4 of these studies also reported ORR in RAM + DTX ICI naïve patients (30, 32, 38, 41) studies (**Figure 2C**). In the 4 studies that reported outcomes with RAM + DTX in the ICI pre-treated groups only (33, 34, 39, 40), the ORR ranged from 32.5% (95% CI: NR) to 60.0% (95% CI: 38.5-81.4) (33, 39). In the 4 remaining studies, which reported results from both RAM + DTX ICI pre-treated and RAM + DTX ICI naïve groups (30, 32, 38, 41), the ORR was numerically higher in the RAM + DTX ICI pre-treated groups (38.9%, 20.9%, 57.1%, and 42.6%) compared with the respective RAM + DTX ICI naïve groups (19.0%, 18.3%, 20.0%, and 17.7%) (**Figure 2C**). Across all 8 studies, the ORR ranged from 20.9% (95% CI: NR) (32) to 60.0% (95% CI: 38.5-81.4) (39) in the RAM + DTX ICI pre-treated groups and from 17.7% (95% CI: NR) (41) to 20.0% (95% CI: 5.7-43.7) (38) in the RAM + DTX ICI naïve groups (**Figure 2C**).

#### 3.3.4 Disease control rate

DCRs were reported in 6 of the 10 studies included in the efficacy analysis (30, 33, 34, 38–40). Four studies reported outcomes in the RAM + DTX ICI pre-treated groups alone (33, 34, 39, 40), whereas the remaining 2 studies reported DCRs in both the RAM + DTX ICI pre-treated and ICI-naïve groups (30, 38). Overall, the DCR ranged from 62.4% (95% CI: NR) to 90.0% (95% CI: 76.9-100.0) (33, 39) in the RAM + DTX ICI pre-treated groups and from 57.1% (95% CI: 34.0-78.2) to 75.0% (95% CI: 50.9-91.3) (30, 38) in the RAM + DTX ICI naïve groups (**Figure 2D**).

### 3.4 Safety

Safety data were derived from 8 studies for a total of 493 RAM + DTX ICI pre-treated patients and 341 RAM + DTX ICI-naïve patients (**Table 5**) (30, 32–34, 39, 40, 43, 44). All 8 studies included in the analysis reported any-grade AEs from the RAM + DTX ICI pre-treated group only (30, 32–34, 39, 40, 43, 44), and 3 studies reported any-grade AEs in both the RAM + DTX ICI naïve group and RAM + DTX ICI treated group (**Table 5**) (30, 32, 43).

**Table 5:** Summary of safety outcomes of RAM + DTX in the ICI pre-treated and ICI naïve groups.

Adverse event, n (%)		Harada, 2019 (30)		Kato, 2020 (32)		Chen, 2022^1^ (43)		Shiono, 2019 (39)	Brueckl, 2020^2^ (34)	Brueckl, 2021^2^ (33)	Ishida, 2021^2^ (40)	Reckamp, 2022^2^ (44)	Pooled AEs	
		ICI pre-treated (n=18)	ICI naïve (n=21)	ICI pre-treated (n=77)	ICI naïve (n=94)	ICI pre-treated (n=172)	ICI naïve (n=226)	ICI pre-treated (n=20)	ICI pre-treated (n=67)	ICI pre-treated (n=77)	ICI pre-treated (n=18)	ICI pre-treated (n=44)	ICI pre-treated N1 = 493^3^ N2 = 321^5^	ICI naïve N1 = 341^4^ N2 = 115^6^
Neutropenia	Any grade	6 (33.0)	12 (57.0)	—	—	7 (4.1)	20 (8.9)	3 (15.0)	—	—	—	—	53 (10.8)	32 (9.4)
	Grade ≥3	1 (5.6)	9 (42.9)	—	—	—	—	3 (15.0)	8 (11.9)	12 (15.6)	3 (16.7)	14 (31.8)	41 (12.8)	9 (7.8)
Febrile neutropenia	Any grade	0	3 (14.0)	—	—	12 (7.0)	22 (9.3)	1 (5.0)	—	—	—	—	19 (3.9)	25 (7.3)
	Grade ≥3	0	3 (14.0)	—	—	—	—	1 (5.0)	—	3 (3.9)	—	3 (6.8)	7 (2.2)	3 (2.6)
Stomatitis	Any grade	—	—	16 (20.8)	20 (21.2)	17 (9.9)	27 (12.0)	4 (20)	—	—	—	—	42 (8.5)	47 (13.8)
	Grade ≥3	—	—	4 (5.2)	3 (3.2)	—	—	1 (5.0)	4 (6.0)	1 (1.3)	—	—	10 (3.1)	3 (2.6)
Vasculitis	Any grade	—	—	—	—	—	—	—	—	—	—	—	1 (0.2)	0
	Grade ≥3	—	—	—	—	—	—	—	—	—	1 (5.6)	—	1 (0.3)	0
Fatigue	Any grade	14 (78.0)	11 (52.0)	—	—	—	—	7 (35.0)	—	—	—	—	29 (5.9)	11 (3.2)
	Grade ≥3	0	0	—	—	—	—	0	—	5 (6.5)	—	3 (6.8)	8 (2.5)	0
Proteinuria	Any grade	8 (44.0)	10 (48.0)	—	—	5 (2.9)	10 (4.4)	—	—	—	—	—	13 (2.6)	20 (5.9)
	Grade ≥3	0	0	—	—	—	—	—	—	—	—	—	0	0
Epistaxis	Any grade	2 (11.0)	3 (14.0)	—	—	10 (5.8)	24 (10.6)	7 (35.0)	—	—	—	—	19 (3.9)	27 (7.9)
	Grade ≥3	0	0	—	—	—	—	0	—	—	—	—	0	0
Hematothorax	Any grade	—	—	—	—	—	—	—	—	—	—	—	1 (0.2)	0
	Grade ≥3	—	—	—	—	—	—	—	1 (1.5)	—	—	—	1 (0.3)	0
Hypertension	Any grade	1 (5.6)	4 (19.0)	—	—	6 (3.5)	10 (4.2)	—	—	—	—	—	9 (1.8)	14 (4.1)
	Grade ≥3	0	1 (4.8)	—	—	—	—	—	—	—	—	2 (4.5)	2 (0.6)	1 (0.9)

^1^This study only reported events of any grade for ICI pre-treated and naïve groups; ^2^These studies only reported grade ≥3 adverse events and were also used to calculate cumulative any-grade AEs.^3,4^N1 is the percentage of any-grade AEs were calculated based on the cumulative safety population for the RAM + DTX ICI pre-treated group (493 patients) and RAM + DTX ICI naïve group (341 patients).^5,6^N2 is the percentages of grade ≥3 AEs were calculated based on the 321 patients in the RAM + DTX ICI pre-treated group and 115 patients in the RAM + DTX ICI naïve group. This difference was because the Chen et al. study did not contribute grade ≥3 AEs to the data pooling due to the absence of published data in the manuscript.

Different number of patients for the safety and efficacy analysis across studies.

DTX, Docetaxel; ICI, Immune checkpoint inhibitor; n, Number of patients; RAM, Ramucirumab.

Grade ≥3 AE data were derived from 7 studies for a total of 321 RAM + DTX ICI pre-treated patients and from 2 studies for a total of 115 patients in the RAM + DTX ICI naïve group (30, 32–34, 39, 40, 44). Of these 7 studies, 4 reported only grade ≥3 AEs (33, 34, 40, 44). For the purpose of this analysis, we focused on hematologic AEs, including neutropenia and febrile neutropenia; two nonhematologic AEs: fatigue and stomatitis; and AEs of special interest for antiangiogenic treatment, including bleeding events, hypertension, and proteinuria.

**Figure 3:**
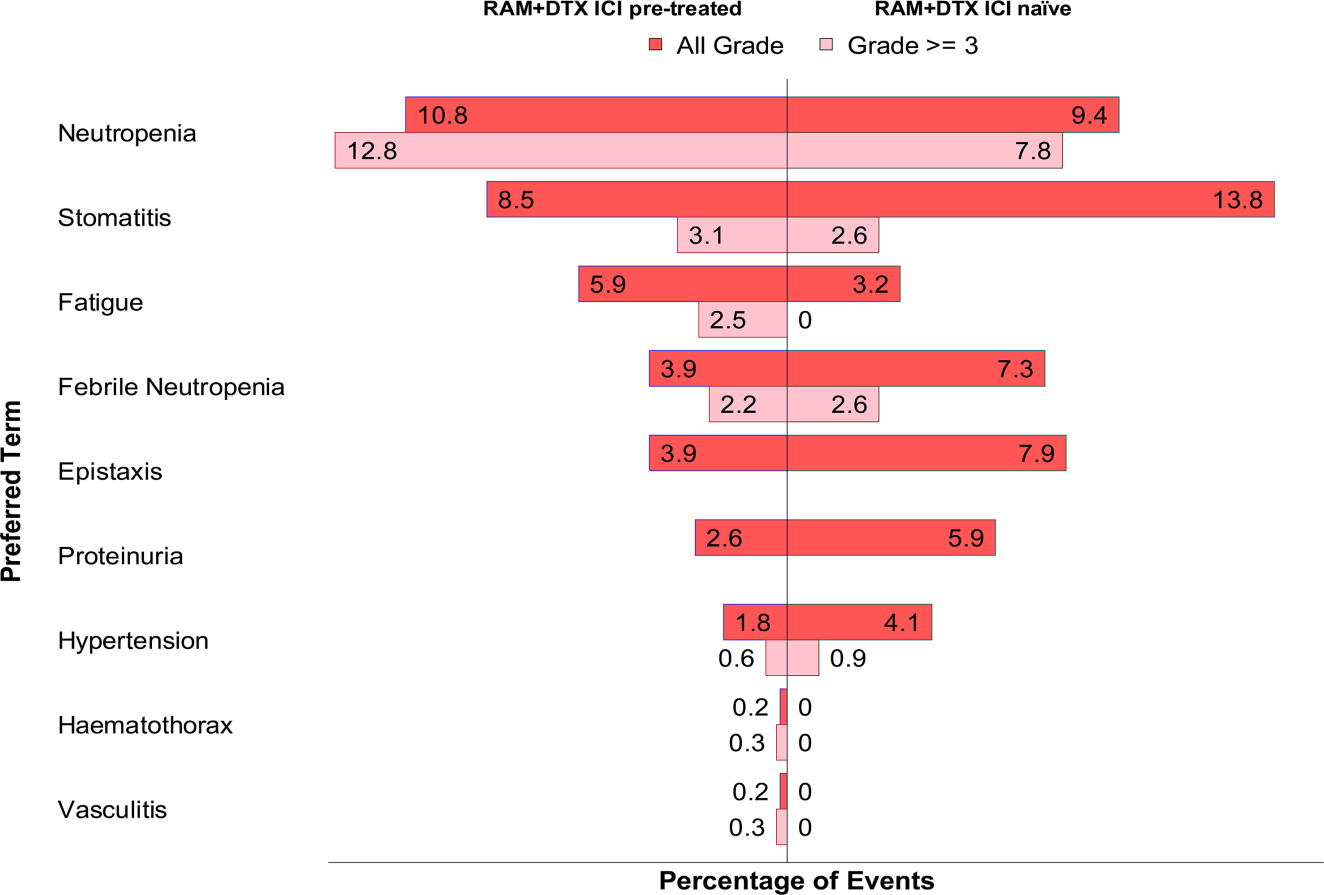
Pooled adverse events of any grade and grade ≥3 in RAM + DTX ICI pre-treated and naïve groups. The percentage of any-grade AEs were calculated based on the cumulative safety population for the RAM + DTX ICI pre-treated group (493 patients) and RAM + DTX ICI naïve group (341 patients), while the percentages of grade ≥3 AEs were calculated based on the 321 patients in the RAM + DTX ICI pre-treated group and 115 patients in the RAM + DTX ICI naïve group. This difference was because the Chen et al. study did not contribute grade ≥3 AEs to the data pooling due to the absence of published data in the manuscript. DTX, Docetaxel; ICI, Immune checkpoint inhibitor; RAM, Ramucirumab.

The percentage of any-grade AEs were calculated based on the cumulative safety population for the RAM + DTX ICI pre-treated group (493 patients) (30, 32–34, 39, 40, 43, 44) and RAM + DTX ICI naïve group (341 patients) (30, 32, 43), while the percentages of grade ≥3 AEs were calculated based on the 321 patients in the RAM + DTX ICI pre-treated group (30, 32–34, 39, 40, 44) and 115 patients in the RAM + DTX ICI naïve group (30, 32). This difference was because the Chen et al. study did not contribute grade ≥3 AEs to the data pooling due to the absence of published data in the manuscript (43). Neutropenia of any grade was reported in 53 (10.8%) patients in the RAM + DTX ICI pre-treated group (30, 33, 34, 39, 40, 43, 44) and in 32 (9.4%) patients in the RAM + DTX ICI naïve group (30, 43). Grade ≥3 neutropenia was reported in 41 (12.8%) patients in the RAM + DTX ICI pre-treated group (30, 33, 34, 39, 40, 44) and in 9 (7.8%) patients in the RAM + DTX ICI naïve group (**Figure 3**; **Table 5**) (30).

Febrile neutropenia of any grade was reported in 19 (3.9%) patients in the RAM + DTX ICI pre-treated group (30, 33, 39, 43, 44) and in 25 (7.3%) patients in the RAM + DTX ICI naïve group (30, 43). Grade ≥3 febrile neutropenia was reported in 7 (2.2%) patients in the RAM + DTX ICI pre-treated group (33, 39, 44) and in 3 (2.6%) patients in the RAM + DTX ICI naïve group (**Figure 3**; **Table 5**) (30).

The incidence of neutropenia of any grade in the RAM + DTX ICI pre-treated group was comparable to the RAM + DTX ICI naïve group in the two studies where both treatment groups were evaluated (**Table 5**). Similarly, the incidence and severity of febrile neutropenia were not higher in the RAM + DTX ICI pre-treated group (**Table 5**).

Stomatitis of any grade was reported in 42 (8.5%) patients in the RAM + DTX ICI pre-treated group (32–34, 39, 43) and in 47 (13.8%) patients in the RAM + DTX ICI naïve group (32, 43). Grade ≥3 stomatitis was reported in 10 (3.1%) patients in the RAM + DTX ICI pre-treated group (32–34, 39) and in 3 (2.6%) patients in the RAM + DTX ICI naïve group (**Figure 3**; **Table 5**) (32).

Fatigue of any grade was reported 29 (5.9%) patients in the RAM + DTX ICI pre-treated group (30, 33, 39, 44) and in 11 (3.2%) patients in the RAM + DTX ICI naïve group (30). Grade ≥3 fatigue was reported in 8 (2.5%) patients in the RAM + DTX ICI pre-treated group (33, 44), and no cases were reported in the RAM + DTX ICI naïve group (**Figure 3**; **Table 5**).

Among AEs of special interest, bleeding or epistaxis of any grade was reported in 19 (3.9%) patients in the RAM + DTX ICI pre-treated group (30, 39, 43) and in 27 (7.9%) patients in the RAM + DTX ICI naïve group (30, 43); no cases of grade ≥3 epistaxis were reported in either group.

The overall incidence of hypertension of any grade in the RAM + DTX ICI pre-treated and RAM + DTX ICI naïve groups was 9 (1.8%) and 14 (4.1%), respectively (30, 43, 44). The incidence of grade ≥3 hypertension in the RAM + DTX ICI pre-treated and RAM + DTX ICI naïve groups was 2 (0.6%) patients and 1 (0.9%) patient, respectively (**Figure 3**; **Table 5**).

Proteinuria of any grade was reported in 13 (2.6%) patients in the RAM + DTX ICI pre-treated group and in 20 (5.9%) patients in the RAM + DTX ICI naïve group (30, 43). No cases of grade ≥3 proteinuria were reported in either group (**Figure 3**; **Table 5**).

## 4 Discussion

The treatment landscape for patients with NSCLC that has progressed after front-line therapies has changed dramatically with the approval and availability of ICIs targeting coinhibitory molecules including anti-PD-1, anti-PD-L1, or anti-CTLA-4 (1–6). The introduction of ICIs in the first-line setting has resulted in significant improvement in efficacy outcomes; however, it has also generated a certain degree of complexity and uncertainty pertaining to the optimal treatment sequence for patients with disease progression during or after immunotherapy, mainly because the currently recommended treatment options for subsequent therapy, including the REVEL regimen, were investigated prior to the approval of ICIs for immunotherapy naïve patients (13, 14, 18–21). Thus, assessment of efficacy and safety for the combination of ramucirumab plus docetaxel after administration of ICIs provides important clinical information in support of its role in sequencing strategies after ICIs.

We analyzed the available information, derived mostly from retrospective observational studies, on the efficacy and safety of ramucirumab plus docetaxel in patients with metastatic NSCLC who had received prior ICIs with the aim of advancing the understanding of the clinical benefit and safety profile of ramucirumab plus docetaxel in the post-immunotherapy setting. To the best of our knowledge, we report the first SLR on the efficacy and safety of ramucirumab plus docetaxel in patients with NSCLC whose disease had progressed after treatment with ICIs either as single agents or in combination with chemotherapy.

We performed a weighted analysis of PFS and OS to adjust for the differing sample sizes across the studies included in the efficacy analysis. Based on this analysis, a numerically longer pooled weighted median PFS was observed with RAM + DTX ICI pre-treated patients compared with RAM + DTX ICI-naïve patients. The signal of improvement of median PFS was observed across all studies included in the efficacy analysis for the RAM + DTX ICI pre-treated group, although statistical significance for the treatment effect was reported in only 2 studies (30, 31). In contrast to PFS, the pooled weighted median OS was numerically longer in the RAM + DTX ICI naïve group compared with the RAM + DTX ICI pre-treated group. These results reflect the variations in median OS across the studies included in the pooled weighted analysis. Among the 5 studies that evaluated OS with ramucirumab plus docetaxel in both ICI pre-treated and ICI naïve groups, 3 reported an improvement in median OS in the RAM + DTX ICI pre-treated group compared with the ICI naïve group (30, 32, 41) while the remaining 2 studies reported numerically longer OS in the RAM + DTX ICI naïve group compared with the ICI pre-treated group (31, 38). These discrepancies in outcomes across studies are likely a result of several factors including imbalances in patient baseline and disease characteristics, differences in lines of treatment with ramucirumab plus docetaxel being administered in second line versus third and later lines, differences in timing between ICI administration and initiation of ramucirumab plus docetaxel therapy, and due to small sample sizes. It is also important to emphasize the contribution of variations in the proportion of patients with *EGFR* mutations across all the studies included in our pooled efficacy analysis. Thus, while 3 studies (30, 32, 38) included patients with mutant *EGFR*-positive lung adenocarcinoma who were treated with ramucirumab plus docetaxel, two others (34, 40) excluded patients who had *EGFR* or *ALK* alterations. In contrast, the remaining studies either reported mutation status for the total ramucirumab plus docetaxel treated populations and did not separate characteristics by ICI pre-treated or naïve (31, 43) or did not specify the type of oncogenic alteration (*EGFR* or *ALK*) (33, 36, 39, 41).

Only a few studies included in the efficacy analysis of this SLR included a control cohort of single-agent chemotherapy administered after ICIs (32, 38, 41). Therefore, a formal efficacy analysis of ramucirumab plus docetaxel relative to single-agent docetaxel after immunotherapy was not performed. However, in the retrospective observational study conducted by Kato et al, where a propensity score was used to correct for imbalances in patient characteristics across the ICI pre-treated and the ICI naïve cohorts, median OS in the subgroup of patients treated with single-agent docetaxel (n=102) or ramucirumab plus docetaxel (n=62) after anti-PD-1 treatment was 9.0 months and 17.5 months, respectively (32). Similarly, the retrospective study by Tozuka et al. in patients who had received prior anti-PD-(L)1 antibody therapy reported a median OS of 8.6 months in the cohort treated with single-agent docetaxel (n=18) and 19.8 months in the cohort treated with ramucirumab plus docetaxel (n=21). Additionally, a significant PFS improvement with ramucirumab plus docetaxel compared to docetaxel was reported (median PFS: 5.9 vs 2.8 months, HR: 0.43; 95% CI: 0.20-0.96; P=0.03) (41).

The combination of platinum-based chemotherapy and ICIs is currently the most widely used therapeutic option for the treatment of newly diagnosed patients with metastatic NSCLC without driver alterations (52). Results from real-world studies indicate that ramucirumab plus docetaxel is a widely used second-line therapy in NSCLC (53). Among the studies included in our analysis, only Brueckl et al. (2021) (33) and Ishida et al. (40) investigated the clinical benefit of second-line ramucirumab plus docetaxel after progression during or after chemoimmunotherapy. Of note, approximately two-thirds of patients in both studies had received prior treatment with pembrolizumab plus pemetrexed and platinum-based chemotherapy (33, 40). Both studies reported efficacy outcomes with ramucirumab plus docetaxel comparable to those reported in the REVEL trial with the exception of higher response rates. High response rates in the RAM + DTX ICI pre-treated group were also reported by other studies included in the efficacy analysis (30, 32–34, 38–41). Although OS is the gold standard to establish treatment efficacy, ORR represents an important clinical endpoint given the high symptom burden experienced by patients with lung cancers (54). Results from the VARGADO trial investigating the combination of nintedanib plus docetaxel in the post-ICI setting also reported improved ORR (58.0%) and median PFS (5.5 months) in patients with NSCLC receiving second-line nintedanib plus docetaxel after chemoimmunotherapy (55).

Prior response to immunotherapy and the timing between ICI treatment and initiation of the subsequent treatment may influence clinical responses to the next line of therapy. Among the studies included in the efficacy analysis of this SLR, few investigated the potential association between prior ICI treatment and improved efficacy outcomes with ramucirumab plus docetaxel. Prior treatment with ICIs was found to be an independent predictive factor for improvement in PFS with ramucirumab plus docetaxel by Harada et al. (30). Yoshimura et al. also demonstrated that prior immunotherapy was an independent prognostic factor for prolonged PFS, but not OS, with ramucirumab plus docetaxel (HR: 0.59; 95% CI: 0.35-0.98; *P*=0.041) (31). The potential association between response to prior immunotherapy and subsequent response to ramucirumab plus docetaxel was also investigated. Ishida et al. demonstrated that clinical benefit from prior ICI therapy, defined as a PFS of ≥8.8 months, was associated with a significantly longer PFS with ramucirumab plus docetaxel compared with the group of patients with a PFS of <8.8 months (HR: 0.12; 95% CI: 0.03-0.48; *P*=0.003) (40). The timing of administration of ramucirumab plus docetaxel after ICI treatment was investigated by Yoshimura et al. A trend suggesting prolonged PFS (HR: 0.54; 95% CI: 0.21-1.40; *P*=0.202) and OS (HR: 0.49; 95% CI: 0.22-1.10; *P*=0.079) was observed when ramucirumab plus docetaxel was administrated consecutively with ICI treatment (31). Taken together, these findings support the role of ramucirumab plus docetaxel in post-immunotherapy sequencing strategies.

The significant association between prior ICIs and favorable clinical outcomes with ramucirumab plus docetaxel may be explained, at least in part, by a role of ramucirumab in overcoming resistance to ICIs, a critical contributing factor that limits the efficacy of immune checkpoint blockade therapy in many patients (56, 57). Although several aspects of the underlying mechanisms responsible for resistance to ICIs have yet to be identified, emerging evidence supports a causal role of tumor extrinsic factors including immunosuppressive signals emanating from the tumor microenvironment (58). In addition to promoting angiogenesis, VEGF exerts important immunosuppressive effects on the tumor microenvironment. VEGF-induced abnormal neovascularization not only limits the access of tumor-directed cytotoxic T cells but also stimulates the recruitment of immunosuppressive cells, including myeloid-derived suppressor cells and regulatory T cells, and inhibits dendritic cell maturation, which ultimately leads to decreased activation of antigen-specific cytotoxic T cells (59–61). Therefore, targeting angiogenesis represents a rational approach to hinder immunosuppressive signals within the tumor microenvironment and restore antitumor cytotoxic T-cell responses. The immunomodulatory effects of ramucirumab may also explain the antitumor effects of ramucirumab combinations with anti-PD-(L)1 antibodies. Preclinical studies support this possibility. In a recently published study, the combination of DC101, a mouse surrogate of ramucirumab, with an anti-PD-1 antibody induced tumor regression and immunological memory in EMT6-LM2 and MC38 murine tumor models (62). Furthermore, the recently reported results from the phase 2 S1800A study of Lung-MAP demonstrated an improvement in OS (both median OS and HR), albeit no difference in ORR and PFS, with ramucirumab plus pembrolizumab compared with other standard-of-care options in patients with metastatic NSCLC whose disease had progressed on prior chemoimmunotherapy (63). Based on these results, a phase 3 trial investigating the combination of ramucirumab plus pembrolizumab in the same patient population is currently enrolling (NCT05633602) (64). An early efficacy signal has also been reported with ramucirumab in combination with atezolizumab in a heavily pre-treated NSCLC patient population (65), while another study investigating the combination of ramucirumab plus nivolumab in patients previously treated with ICIs and chemotherapy is currently ongoing (NCT03527108) (66).

A total of 11 studies were excluded from the efficacy analysis because they did not meet the inclusion criteria. However, results from these excluded studies also indicated that ramucirumab plus docetaxel was a safe and efficacious treatment after prior treatment with ICIs (35, 37, 42, 45–47, 67–70). Five of these were real-world studies that used electronic health record-derived databases were not included in the efficacy analysis because they did not meet the SLR inclusion criteria (45–47, 71) or they had a high percentage of missing data (e.g., tumor response assessment) and therefore had a high potential for misclassification and residual confounding bias (72). However, results from most of these studies suggest a trend towards improved efficacy outcomes with ramucirumab plus docetaxel administered after ICIs. In a small cohort of patients treated with second-line ramucirumab plus docetaxel after chemoimmunotherapy, real-world ORR and DCR were 37.5% and 75.0%, respectively (46). In an additional study by Clarke et al, ramucirumab plus docetaxel administered as a second- or third-line therapy after chemotherapy plus ICIs was associated with numerically higher real-world ORR (40.9% vs 30.4; *P*=0.21). Moreover, a statistically significant improvement in real-world DCR was observed when compared to ramucirumab plus docetaxel given after other non–ICIs (80.7% vs 54.4%; *P*<0.01) (45). Furthermore, in a cohort of patients treated with third-line ramucirumab plus docetaxel after ICI treatment, median real-world PFS (measured from the start of third-line ramucirumab plus docetaxel) was 3.6 months (range: 3.0-4.6 months) while median real-world OS (measured from the start of first-line therapy) was 19 months (range: 15.7-23.7 months) (47). The TREAT-LUNG study, a large retrospective observational study that collected data from 3 electronic health record-derived databases, investigated efficacy outcomes of second- or third-line therapy with ramucirumab plus docetaxel administered after prior chemotherapy plus ICIs. Study results showed that, after adjustment for baseline variables, differences in response rates, PFS, and OS were not statistically significant but trended in favor of ramucirumab plus docetaxel as observed in the REVEL trial (72). Taken together, with the limitations inherent to real-world data, these studies are supportive of a signal for increased activity of ramucirumab plus docetaxel in the post-immunotherapy setting.

A recently disclosed single-arm, multicenter, prospective phase 2 study of ramucirumab plus docetaxel following chemoimmunotherapy (SCORPION) in patients with metastatic NSCLC was not included in the SLR analysis as it was reported outside the window of literature search. This study demonstrated median PFS and median OS of 6.5 months and 17.5 months, respectively. ORR and DCR were 34.4% and 81.3%, respectively (73). No new safety signals were observed (73).

The descriptive safety analyses reported in this SLR support the conclusion that the select AEs observed with ramucirumab plus docetaxel administered after immunotherapy were similar in incidence and severity to those reported in the REVEL trial. Importantly, no new safety signals or additive toxicities emerged with ramucirumab plus docetaxel when administered after ICI treatment, including in the only controlled prospective study in the analysis (44). Notably, the retrospective study conducted by Harada et al. reported a higher incidence of pneumonitis in Japanese patients who had received ICIs before ramucirumab plus docetaxel; however, a causal relationship with ramucirumab plus docetaxel was not established (30). On the other hand, in the post marketing study by Chen et al, the incidence of pneumonitis of any grade in ICI-exposed and ICI-naïve patients was 1.2% and 0.9%, respectively (43). No cases of pneumonitis were observed or reported in the remaining studies. The safety analyses presented here must be interpreted with caution because most of the studies included in the safety analysis were retrospective and therefore underreporting of AEs cannot be ruled out.

Overall, the results presented in this SLR should be interpreted in the context of limitations inherent to the retrospective nature of most studies included in the analysis. To offset some limitations and decrease the heterogeneity of the data across studies, we included only studies free of bias that reported at least 2 efficacy endpoints with at least one being a time-to-event endpoint and performed weighted analyses to control for sample size. Despite the limitations, we believe that in the absence of controlled randomized clinical trials, the results present here provide valuable clinical information to complement current guideline recommendations for the use of ramucirumab plus docetaxel as a subsequent therapy in metastatic NSCLC.

In conclusion, the results of this SLR support the combination of ramucirumab plus docetaxel as an effective and safe subsequent therapy for the treatment of patients with metastatic NSCLC with disease progression regardless of prior treatment with ICIs.

## Data availability statement

The original contributions presented in the study are included in the article/**Supplementary Material**. Further inquiries can be directed to the corresponding author.

## Author contributions

CV-G, MTR, SC, TP, and MR were involved in the conception and design of the work. EG, SC, and MR were involved in the acquisition of data for the work. SC, TP, and MR were involved in the analysis of the data for the work. MTR, SC, and TP were involved in drafting of the work. All authors were involved in the interpretation of the data for the work as well as critical revision to the work.
