# Supplementary material for: Clinical outcomes of ramucirumab plus docetaxel in the treatment of patients with non-small cell lung cancer after immunotherapy: a systematic literature review
Source: Front Oncol. 2023 Sep 4;13:1247879. doi: 10.3389/fonc.2023.1247879 (PMC10507469; doi:10.3389/fonc.2023.1247879)
Supplement: Supplementary file 1 [file Table_1.docx]

Supplementary Material

Clinical outcomes of ramucirumab plus docetaxel in the treatment of patients with non-small cell lung cancer after immunotherapy: a systematic literature review

**Edward B. Garon, Carla Visseren-Grul, Maria Teresa Rizzo, Tarun Puri, Suresh Chenji, Martin Reck***

***Correspondence:** Martin Reck (M.Reck@lungenclinic.de)

# Supplementary data

**1.1 Search strategy**

The following search criteria were applied:

((non-small cell lung cancer) OR (non-small-cell lung carcinoma) OR (nonsmall cell lung cancer) OR (nonsmall cell lung carcinoma) OR (NSCLC) OR (lung cancer) OR (lung neoplasms) OR (lung malignan*) OR (lung tumours) OR (lung tumors)) AND ((Docetaxel) OR (DTX)) AND ((Ramucirumab) OR (RAM) OR (antiangiogenic*) OR (VEGF inhibitor*) OR (anti-VEGF) OR (Anti-VEGF receptor-2) OR (Anti-VEGFR2)) AND ((Prior) OR (Previous*) OR (Following) OR (Post)) AND ((Immunotherap*) OR (immuno-oncology) OR (IO) OR (Immune checkpoint inhibitor) OR (Nivolumab) OR (Pembrolizumab) OR (Atezolizumab) OR (ICI) OR (anti-PD-1) OR (anti‑PD‑L1)) AND ((Chemo*) OR (platinum)) NOT ((CTLA-4) OR (anti-CTLA-4)).

This was a simple systematic literature review. A formal protocol was not created, and it was not registered on PROSPERO.

# Supplementary figures and tables

## Supplementary tables

**Supplementary Table 1. Risks of bias assessment of controlled studies (Newcastle-Ottawa Scale** **assessment tool).**

|  | | | **Harada,**  **2019 (1)** | **Yoshimura, 2019 (2)** | **Kato,**  **2020 (3)** | **Tozuka,  2020 (4)** | **Nishimura,**  **2022 (5)** | **Chen,**  **2022 (6)** |
| --- | --- | --- | --- | --- | --- | --- | --- | --- |
| Selection | Representativeness of the exposed cohort | a) Truly representative of the average prior line of therapy in the community* | a1 | a1 | a1 | a1 | a1 | a1 |
|  |  | b) Somewhat representative of the average prior line of therapy in the community* |  |  |  |  |  |  |
|  |  | c) Selected group of users, eg, nurses, volunteers |  |  |  |  |  |  |
|  |  | d) No description of the derivation of the cohort |  |  |  |  |  |  |
|  | Selection of the nonexposed cohort | a) Drawn from the same community as the exposed cohort* | a1 | a1 | a1 | a1 | a1 | a1 |
|  |  | b) Drawn from a different source |  |  |  |  |  |  |
|  |  | c) No description of the derivation of the nonexposed cohort |  |  |  |  |  |  |
|  | Ascertainment of exposure | a) Secure record (eg, surgical records)* | a1 | a1 | a1 | a1 | a1 | a1 |
|  |  | b) Structured interview* |  |  |  |  |  |  |
|  |  | c) Written self-report |  |  |  |  |  |  |
|  |  | d) No description |  |  |  |  |  |  |
|  | Demonstration that outcome of interest was not present at start of study | a) Yes* | a1 | a1 | a1 | a1 | a1 | a1 |
|  |  | b) No |  |  |  |  |  |  |
| Comparability | Comparability of cases and controls on the basis of the design or analysis | a) Study controls for RAM + DTX ICI pretreated, RAM + DTX ICI naïve* | a1b1 | a1b1 | a1b1 | a1b1 | a1b1 | a1b1 |
|  |  | b) Study controls for any additional factor of at least one prior line of therapy* |  |  |  |  |  |  |
| Outcome | Assessment of outcome | a) Independent blind assessment* | b1 | b1 | b1 | b1 | b1 | b1 |
|  |  | b) Record linkage* |  |  |  |  |  |  |
|  |  | c) Self-report |  |  |  |  |  |  |
|  |  | d) No description |  |  |  |  |  |  |
|  | Was follow-up long enough for outcomes to occur? | a) Yes* | a1 | a1 | a1 | a1 | a1 | a1 |
|  |  | b) No |  |  |  |  |  |  |
|  | Adequacy of follow‑up of cohorts | a) Complete follow‑up – all subjects accounted for* | a1 | a1 | a1 | a1 | a1 | a1 |
|  |  | b) Subjects lost to follow‑up unlikely to introduce bias – small number lost ≤20% follow‑up* |  |  |  |  |  |  |
|  |  | c) Follow‑up rate >20% and no description of those lost |  |  |  |  |  |  |
|  |  | d) No statement |  |  |  |  |  |  |
| Total Score | |  | 9 | 9 | 9 | 9 | 9 | 9 |

Allocation of stars as per rating sheet (7). Note: A study can be awarded a maximum of 1 star for each numbered item within the Selection and Outcome categories. A maximum of 2 stars can be given for Comparability. Total score is the sum of number of sub-categories met (which is indicated by symbol 1) assessed for each study.

DTX, Docetaxel; ICI, Immune checkpoint inhibitor; RAM, Ramucirumab.

**Supplementary Table 2. Risks of bias assessment of non-controlled studies (National Institutes of Health assessment tool).**

| **Assessment question** | **Response** | **Shiono, 2019 (8)** | **Brueckl, 2020 (9)** | **Brueckl, 2021 (10)** | **Dawar, 2021 (11)** | **Ishida, 2021 (12)** | **Reckamp, 2022 (13)** |
| --- | --- | --- | --- | --- | --- | --- | --- |
| 1. Was the study question or objective clearly stated? | Yes | Yes | Yes | Yes | Yes | Yes | Yes |
|  | No |  |  |  |  |  |  |
|  | Other (CD, NR, NA) |  |  |  |  |  |  |
| 2. Were eligibility/selection criteria for the study population prespecified and clearly described? | Yes | Yes | Yes | Yes | Yes | Yes | Yes |
|  | No |  |  |  |  |  |  |
|  | Other (CD, NR, NA) |  |  |  |  |  |  |
| 3. Were the participants in the study representative of those who would be eligible for the test/service/intervention in the general or clinical population of interest? | Yes | Yes | Yes | Yes | Yes | Yes | Yes |
|  | No |  |  |  |  |  |  |
|  | Other (CD, NR, NA) |  |  |  |  |  |  |
| 4. Were all eligible participants that met the prespecified entry criteria enrolled? | Yes | Yes | Yes | Yes | Yes | Yes | Yes |
|  | No |  |  |  |  |  |  |
|  | Other (CD, NR, NA) |  |  |  |  |  |  |
| 5. Was the sample size sufficiently large to provide confidence in the findings? | Yes |  | Yes | Yes |  |  | Yes |
|  | No | No |  |  | No | No |  |
|  | Other (CD, NR, NA) |  |  |  |  |  |  |
| 6. Was the test/service/intervention clearly described and delivered consistently across the study population? | Yes | Yes | Yes | Yes | Yes | Yes | Yes |
|  | No |  |  |  |  |  |  |
|  | Other (CD, NR, NA) |  |  |  |  |  |  |
| 7. Were the outcome measures prespecified, clearly defined, valid, reliable, and assessed consistently across all study participants? | Yes | Yes | Yes | Yes | Yes | Yes | Yes |
|  | No |  |  |  |  |  |  |
|  | Other (CD, NR, NA) |  |  |  |  |  |  |
| 8. Were the people assessing the outcomes blinded to the participants’ exposures/interventions? | Yes |  |  |  |  |  |  |
|  | No | No | No | No | No | No | No |
|  | Other (CD, NR, NA) |  |  |  |  |  |  |
| 9. Was the loss to follow-up after baseline 20% or less? Were those lost to follow-up accounted for in the analysis? | Yes |  |  |  |  |  | Yes |
|  | No |  |  |  |  |  |  |
|  | Other (CD, NR, NA) | NR | NR | NR | NR | NR |  |
| 10. Did the statistical methods examine changes in outcome measures from before to after the intervention? Were statistical tests done that provided *P* values for the pre-to-post changes? | Yes | Yes | Yes | Yes | Yes | Yes | Yes |
|  | No |  |  |  |  |  |  |
|  | Other (CD, NR, NA) |  |  |  |  |  |  |
| 11. Were outcome measures of interest taken multiple times before the intervention and multiple times after the intervention (ie, did they use an interrupted time-series design)? | Yes |  |  |  |  |  |  |
|  | No |  |  |  |  |  |  |
|  | Other (CD, NR, NA) | NR | NR | NR | NR | NR | NR |
| 12. If the intervention was conducted at a group level (eg, a whole hospital, a community, etc.) did the statistical analysis take into account the use of individual-level data to determine effects at the group level? | Yes | Yes | Yes | Yes | Yes | Yes | Yes |
|  | No |  |  |  |  |  |  |
|  | Other (CD, NR, NA) |  |  |  |  |  |  |

CD, Cannot determine; NR, Not reported; N/A, Not applicable.

**References**

Harada D, Takata K, Mori S, Kozuki T, Takechi Y, Moriki S, et al. Previous immune checkpoint inhibitor treatment to increase the efficacy of docetaxel and ramucirumab combination chemotherapy. Anticancer Res (2019) 39(9):4987-93. doi: 10.21873/anticanres.13688

Yoshimura A, Yamada T, Okuma Y, Kitadai R, Takeda T, Kanematsu T, et al. Retrospective analysis of docetaxel in combination with ramucirumab for previously treated non-small cell lung cancer patients. Transl Lung Cancer Res (2019) 8(4):450-60. doi: 10.21037/tlcr.2019.08.07

Ryoji Kato HH, Yasutaka Chiba, Eriko Miyawaki, Junichi Shimizu, Tomohiro Ozaki, Daichi Fujimoto, et al. Propensity score–weighted analysis of chemotherapy after PD-1 inhibitors versus chemotherapy alone in patients with non–small cell lung cancer (WJOG10217L). J Immunother Cancer (2020) 8(1):e000350. doi: [10.1136/jitc-2019-000350](https://doi.org/10.1136/jitc-2019-000350)

Tozuka T, Kitazono S, Sakamoto H, Yoshida H, Amino Y, Uematsu S, et al. Addition of ramucirumab enhances docetaxel efficacy in patients who had received anti-PD-1/PD-L1 treatment. Lung Cancer (2020) 144:71-5. doi: 10.1016/j.lungcan.2020.04.021

Nishimura T, Fujimoto H, Okano T, Naito M, Tsuji C, Iwanaka S, et al. Is the efficacy of adding ramucirumab to docetaxel related to a history of immune checkpoint inhibitors in the real-world clinical practice? Cancers (Basel) (2022) 14(12):2970. doi: 10.3390/cancers14122970

Chen Y, Nagaoka S, Katayose T, Sekine N. Safety and effectiveness of ramucirumab and docetaxel: a single-arm, prospective, multicenter, non-interventional, observational, post‑marketing safety study of NSCLC in Japan. Expert Opin Drug Saf (2022) 21(5):691-8. doi: 10.1080/14740338.2022.2023127

Wells GA, Shea B, O’Connell D, Peterson J, Welch V, Losos M, et al. The Newcastle-Ottawa Scale (NOS) for assessing the quality of nonrandomised studies in meta-analyses. Eur J Epidemiol (2011) 25:603-5.

Shiono A, Kaira K, Mouri A, Yamaguchi O, Hashimoto K, Uchida T, et al. Improved efficacy of ramucirumab plus docetaxel after nivolumab failure in previously treated non-small cell lung cancer patients. Thorac Cancer (2019) 10(4):775-81. doi: 10.1111/1759-7714.12998

Brueckl WM, Reck M, Rittmeyer A, Kollmeier J, Wesseler C, Wiest GH, et al. Efficacy of docetaxel plus ramucirumab as palliative second-line therapy following first-line chemotherapy plus immune-checkpoint-inhibitor combination treatment in patients with non‑small cell lung cancer (NSCLC) UICC stage IV. Transl Lung Cancer Res (2021) 10(7):3093-105. doi: [10.21037/tlcr-21-197](https://doi.org/10.21037/tlcr-21-197)

Brueckl WM, Reck M, Rittmeyer A, Kollmeier J, Wesseler C, Wiest GH, et al. Efficacy of docetaxel plus ramucirumab as palliative third-line therapy following second-line immune‑checkpoint-inhibitor treatment in patients with non-small-cell lung cancer stage IV. Clin Med Insights Oncol (2020) 14:1179554920951358. doi: 10.1177/1179554920951358

Dawar R, Gawri K, Rodriguez E, Thammineni V, Saul E, Lima Filho JOO, et al. P01.09 Improved outcomes with ramucirumab & docetaxel in metastatic non-small cell lung cancer after failure of immunotherapy. In: International Association for the Study of Lung Cancer 2020 World Conference on Lung Cancer Singapore, 2021 Jan 28-31; Singapore. J Thorac Oncol (2021) 16(3):S239-S240. doi: 10.1016/j.jtho.2021.01.333

Ishida M, Morimoto K, Yamada T, Shiotsu S, Chihara Y, Yamada T, et al. Impact of docetaxel plus ramucirumab in a second-line setting after chemoimmunotherapy in patients with non-small-cell lung cancer: a retrospective study. Thorac Cancer (2022) 13(2):173-81. doi: [10.1111/1759-7714.14236](https://doi.org/10.1111/1759-7714.14236)

Reckamp KL, Redman MW, Dragnev KH, Minichiello K, Villaruz LC, Faller B, et al. Phase II randomized study of ramucirumab and pembrolizumab versus standard of care in advanced non-small-cell lung cancer previously treated with immunotherapy-Lung-MAP S1800A. J Clin Oncol (2022) 40(21):2295-306. doi: 10.1200/JCO.22.00912
